# A tympanitis-related brain abscess caused by *Helcococcus kunzii* in China: a case report and literature review

**DOI:** 10.1186/s12879-025-10895-6

**Published:** 2025-04-12

**Authors:** Liyan Mao, Zhongju Chen, Chao Xu, Xueman Wang, Lu Gong, Sui Gao, Ziyong Sun, Cui Jian

**Affiliations:** Department of Laboratory Medicine, Tongji Hospital, Tongji Medical College, Huazhong University of Science and Technology, Wuhan, 430030 China

**Keywords:** Tympanitis, Brain abscess, *Helcococcus kunzii*, MALDI-TOF, 16S rRNA sequencing

## Abstract

**Background:**

Infections attributed to *Helcococcus kunzii* are rarely documented, especially in relation to brain abscesses. This study aims to report the first documented case of a brain abscess associated with tympanitis caused by *H. kunzii* in China, alongside a comprehensive review of the existing literature.

**Case presentation:**

We detail the case of a 54-year-old female patient with a history of hypertension, who was diagnosed with tympanitis complicated by a brain abscess. An urgent occipital lobectomy was performed, during which pus was collected for culture analysis. Three distinct colony morphologies were identified through matrix-assisted laser desorption/ionization time of flight mass spectrometry (MALDI-TOF MS) analysis, including *H. kunzii* and two additional anaerobic bacterial species. Subsequent biochemical assays and 16S rRNA gene sequencing corroborated the presence of *H. kunzii*. Antimicrobial susceptibility testing revealed that the isolated *H. kunzii* strain exhibited resistance to erythromycin and clindamycin. The patient was subsequently treated with intravenous antibiotics, specifically ceftriaxone, meropenem, and norvancomycin, resulting in complete recovery.

**Conclusions:**

This case underscores the increasing acknowledgment of *H. kunzii* as a notable pathogen in invasive intracranial infections. It is imperative for clinicians to consider *H. kunzii* in the differential diagnosis of patients presenting with intracranial infections, especially those with a history of tympanitis, to ensure prompt and effective management. The utilization of MS and molecular techniques should be prioritized for the accurate identification of these anaerobic bacteria.

**Supplementary Information:**

The online version contains supplementary material available at 10.1186/s12879-025-10895-6.

## Background

*Helcococcus*, a Gram-positive cocci, was initially characterized in 1993 by Collins and colleagues [1]. This microorganism is a facultative anaerobe and catalase-negative. To date, six species within this genus have been identified, namely *Helcococcus kunzii* [1], *Helcococcus ovis* [2], *Helcococcus pyogenica* [3], *Helcococcus sueciensis* [4], *Helcococcus seattlensis* [5] and *Helcococcus massiliensis* [6]. These species have been isolated from a variety of animal and human specimens, suggesting a potential for zoonotic transmission as well as human infections [7–10]. Notably, *H. kunzii* is the most frequently identified pathogenic species [11]. *H. kunzii* is predominantly regarded as a commensal organism on the skin and is generally considered innocuous [12]. However, in immunocompromised individuals, it can act as an opportunistic pathogen [13, 14]. *H. kunzii* has been implicated in a variety of infections, including infective endocarditis, osteomyelitis, bacteremia, and abscesses at various anatomical sites [15–21].

In this report, we present the first documented case of a tympanitis-related brain abscess caused by *H. kunzii* in China, and we provide a comprehensive review of the existing literature on infections associated with *H. kunzii*.

## Case presentation

In November 2023, a 54-year-old female patient presented with a ten-day history of dizziness, headache, and vomiting. She sought medical attention at our Neurology Department due to the persistence of her symptoms despite rest at home. A computed tomography (CT) scan of the head, performed at a local hospital, identified an intracranial space-occupying lesion. The patient’s medical history includes hypertension, which she managed with self-administered medication of limited efficacy. She reported no history of smoking, alcohol consumption, or other underlying diseases. A timeline shown in Supplemental Fig. 1 in Additional file 1.

Upon admission, the patient exhibited a body temperature of 36.8℃, accompanied by normal pulse and respiratory rates. The recorded blood pressure was 199/104 mmHg. The patient demonstrated an altered mental status, while no abnormalities were observed in the skin or lymph nodes. Pupillary examination revealed equal, round, and reactive pupils with a diameter of 2.5 mm, and visual fields were intact. The neck was supple, and no abnormalities were identified during the cardiac, pulmonary, and abdominal examinations. The patient demonstrated normal limb function and muscle strength, exhibiting physiological rather than pathological reflexes. Laboratory investigations indicated elevated inflammatory markers, including leukocytes (10.55 × 10^9/L), neutrophils (6.94 × 10^9/L), and monocytes (0.8 × 10^9/L). The dynamic changes in these indicators during hospitalization are depicted in Supplemental Fig. 2.

**Fig. 1:**
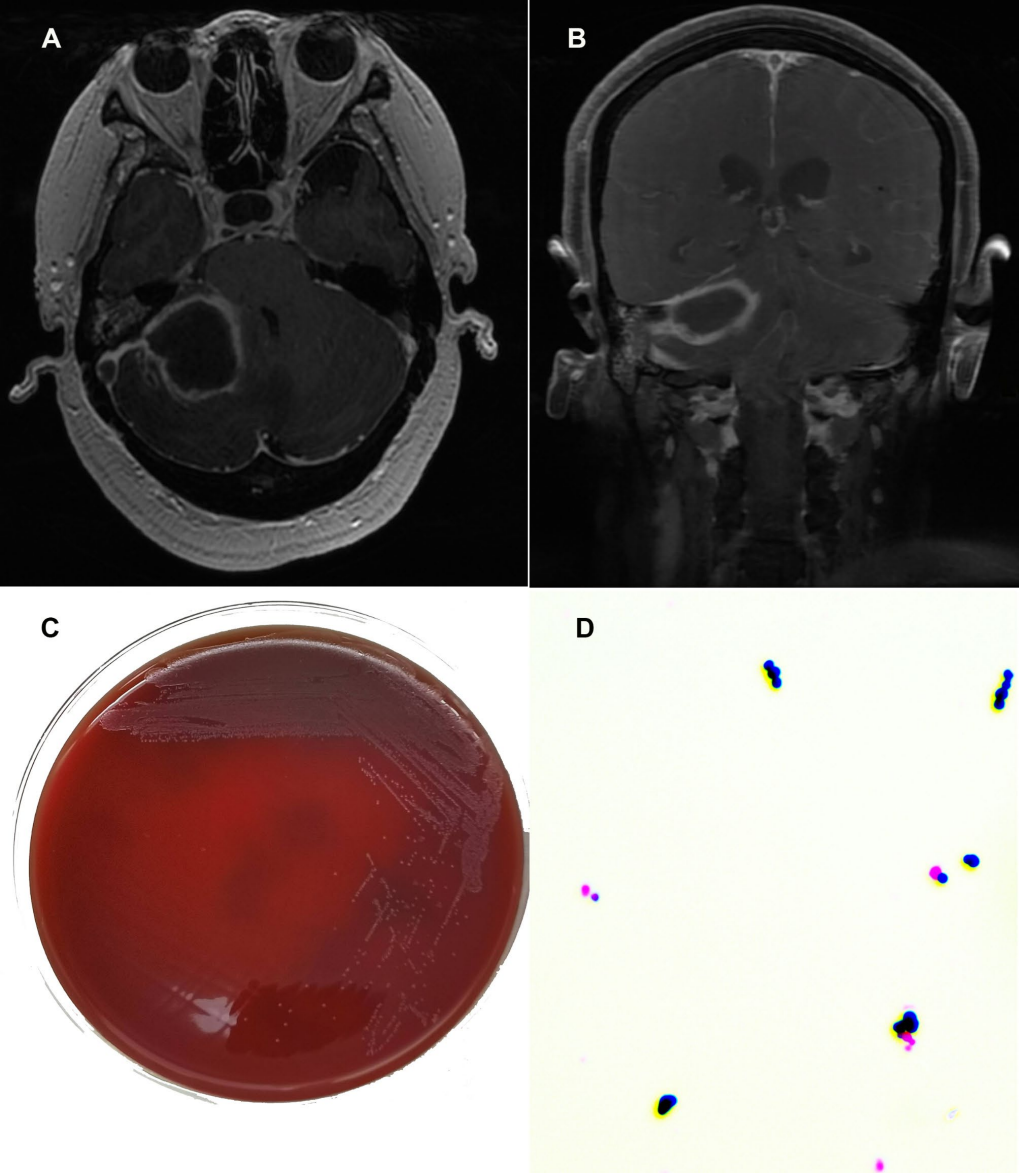
**Imaging and microbiologic characteristics of the patient.** (**A**). and (**B**). Head magnetic resonance imaging scan. (**C**). 1 Culture colonies of *Helcococcus kunzii* on sheep blood agar plates for 48 h. (**D**). Gram staining (× 1000 times) of *H.kunzii*

Upon admission, a head magnetic resonance imaging (MRI) scan conducted on November 21, 2023, revealed circumferential enhancement in the right cerebellopontine angle region, suggestive of an infectious lesion, with possibility of abscess formation. The imaging revealed increased enhancement of the falx cerebri and tentorium, along with a filling defect within the right sigmoid sinus, suggesting the possibility of venous thrombosis. Additionally, there were indications of infection in the right middle ear and mastoid, as illustrated in Fig. 1. Clinically, the patient presented with persistent confusion and ongoing episodes of vomiting. A subsequent MRI of the head, conducted on November 23, 2023, demonstrated no significant abnormalities in the bilateral vestibulocochlear nerves. However, a mixed-signal mass was detected in the right cerebellar hemisphere, with enhancement characteristics indicative of a potential abscess with associated hemorrhage. Right-sided tympanitis was reconfirmed, and imaging of the right sigmoid sinus revealed abnormal signals indicative of venous sinus thrombosis, accompanied by supratentorial hydrocephalus. A chest X-ray and echocardiogram showed no significant cardiopulmonary abnormalities. Integrating the clinical presentation, laboratory findings, and imaging results, in conjunction with a neurosurgical consultation, suggested a high likelihood of a brain abscess associated with right-sided middle ear tympanitis and concurrent venous sinus thrombosis. Surgical intervention for abscess drainage was recommended. Empirical antibiotic therapy was initiated with intravenous ceftriaxone at a dosage of 3 g per day.

On the fourth day post-admission, the patient underwent an occipital lobectomy. During the procedure, the dura mater was incised under microscopic visualization, and a cortical puncture approximately 1 cm in depth was made, revealing the abscess capsule. Pus was aspirated for culture, and tissue samples from the walls of the intracranial abscess were collected for pathological examination. Intraoperative observations raised concerns regarding a potential anaerobic infection. Considering the necessity for comprehensive antimicrobial coverage targeting both Gram-positive cocci and β-lactamase-resistant anaerobes, along with meropenem’s enhanced ability to penetrate the blood-brain barrier, empirical treatment was commenced using intravenous meropenem (1 g every 8 h) for a duration of three days pending the availability of culture results. This therapeutic decision was further substantiated by a postoperative elevation in inflammatory markers: the white blood cell count increased from 10.55 × 10⁹/L preoperatively to 16.10 × 10⁹/L, and the neutrophil percentage rose from 65.7 to 91.2% (refer to Supplemental Fig. 2). Microbiological analysis of the pus aspirated intraoperatively, along with the abscess wall tissue, revealed a polymicrobial infection with *H. kunzii* as the predominant isolate. Histopathological analysis indicated necrotic regions within the cerebral tissue, along with the development of microabscesses. The adjacent inflammatory granulation tissue demonstrated significant infiltration by both acute and chronic inflammatory cells, as well as an accumulation of tissue cells. Additionally, mild reactive hyperplasia of certain glial cells was noted. In light of these findings, the antibiotic regimen was modified to include intravenous administration of norvancomycin at a dosage of 0.8 g, administered twice daily. A follow-up CT scan conducted seven days postoperatively revealed postoperative alterations in the abscess, including the absorption of hemorrhage and gas. The patient was discharged in a stable condition two weeks following admission. At the one-month follow-up, no significant abnormal symptoms were observed.

Postoperatively, a purulent specimen extracted from the brain abscess was submitted to the microbiology laboratory. The specimen was inoculated onto 5% sheep blood Columbia agar plates and chocolate agar to facilitate both aerobic and anaerobic bacterial cultures. After incubation at 35 °C for 24 h under aerobic conditions and 48 h under anaerobic conditions, three distinct colony morphologies were observed. Identification was conducted using matrix-assisted laser desorption/ionization time of flight mass spectrometry (MALDI-TOF MS, Autof ms1000, Autobio, China), which identified the colonies as *H. kunzii*,* Peptoniphilus harei*,* and Peptostreptococcus lactolyticus.* On sheep blood agar, *H. kunzii* exhibited superior growth (4 + colony density), significantly surpassing the sparse growth of *P. harei* and *P. lactolyticus* (1 + colony density). Notably, the colonies of *H. kunzii*, the predominant isolate, were characterized by pinpoint size after 24 h of incubation. After 48 h, they developed into small, colorless, transparent colonies with slight alpha-hemolytic activity (Fig. 1). Gram staining indicated that *H. kunzii* is a Gram-positive coccus, typically occurring singly, in pairs, or in short chains, with no spores, capsules, or flagella. The catalase and oxidase assays for this strain yielded negative results. Phenotypic identification via the Vitek2 compact system (BioMérieux, France) corroborated the presence of *H. kunzii*. Comprehensive biochemical data are presented in Supplementary Table 1. Furthermore, 16S rRNA gene sequencing was conducted on the pathogen via Applied Biosystems 3730xl DNA Analyzer (Thermo Fisher Scientific, USA). Subsequent BLAST analysis indicated that the partial sequence (comprising 1465 nucleotides, GenBank accession number PQ727373) demonstrated 98% homology, with 31 nucleotide discrepancies, compared to the *H. kunzii* sequence (NR029237) initially characterized in 1993 [1].

Antimicrobial susceptibility testing (AST) of *H. kunzii* was performed to evaluate its response to penicillin and ceftriaxone using the E-test assay on blood Mueller-Hinton agar. The susceptibilities to additional antimicrobial agents were assessed via disk diffusion assays, as detailed in Supplementary Table 2. In the absence of specific antimicrobial testing guidelines from the Clinical and Laboratory Standards Institute (CLSI) for *Helcococcus*, interpretations were made in accordance with the CLSI M100-ED34 (2024) guidelines established for *Streptococcus* species [22]. According to the CLSI guidelines [22], the strain under investigation exhibited susceptibility to penicillin, vancomycin, cefotaxime, ceftriaxone, linezolid, levofloxacin, and chloramphenicol. Conversely, it demonstrated resistance to erythromycin and clindamycin.

## Discussion

*H. kunzii* was originally isolated from human clinical samples in 1993 [1]. This facultatively anaerobic, catalase-negative Gram-positive coccus demonstrates unique phylogenetic placement within the *Helcococcus* genus, as evidenced by 16 S rRNA divergence from *Aerococcus* species. The taxon honors Lawrence J. Kunz for his foundational work in Gram-positive bacterial systematics. Morphologically, *H. kunzii* grows as paired or clustered cocci, forming translucent-gray colonies (0.5–1 mm diameter) on sheep blood agar within 48 h [13]. Unlike many fastidious anaerobes, it grows equally well under atmospheric (18–22% O₂), microaerophilic (5% CO₂), and anaerobic conditions, producing non-hemolytic or α-hemolytic colonies across these environments [18, 23].

Published case reports of *H. kunzii* infections (summarized in Table 1) show a predilection for elderly males with comorbidities [5, 12, 15–18, 20, 24–28]. Over one-third of cases describe polymicrobial infections involving *staphylococci*, *enterococci*, *Enterobacterales*, or anaerobic bacteria [5, 12, 15, 17, 19, 26, 28]. While initially characterized as a skin commensal of the lower extremities, emerging evidence redefines it as an opportunistic pathogen, particularly in immunocompromised hosts [19]. The clinical spectrum of *H. kunzii* infections spans both localized and systemic manifestations. Notably, diabetic foot ulcers represent a key clinical manifestation [27], where impaired cutaneous integrity and microangiopathy likely facilitate microbial invasion. However, cases in non-diabetic patients with intact immune function demonstrate its pathogenic versatility beyond diabetic populations, including prosthetic joint infections [16] and soft tissue abscesses [29]. Of particular concern is the organism’s invasive potential, with severe manifestations such as infective endocarditis requiring combined antimicrobial and surgical management [12]. Notably, recent reports document neuroinvasive cases in immunocompetent individuals [28], solidifying its classification as a bona fide pathogen rather than an immunocompromise-dependent opportunist.

**Table 1:** Main features of reported cases of *Helcococcus kunzii* infections

Author	Sex/age of patient (years)	Underlying condition(s)	Type of infection	Treatment	Outcome	Other bacteria	Methods of identification
Peel et al., 1997	M, 36	Hypertension, obesity, hypercholesteremia	Infected sebaceous cyst	Flucloxacillin IV and orally for 5 days	Recovery	None	API 20 Strep system
Chagla et al., 1998	F, 57	None	Breast abscess	Cephalexin 0.5 g orally every 8 h	Recovery	None	API 20 S Strep system, 16 S rDNA gene sequencing
Riegel et al., 2003	F, 36	None	Post-surgical foot abscess	Pristinamycin and rifampicin	Recovery	None	Rapid ID 32 Strep system, 16 S rDNA gene sequencing
Woo et al., 2005	M, 41/M, 55	Intravenous-drug use / Intravenous-drug use, alcoholism	Bacteremia / Empyema thoracis	3 weeks of penicillin G and cloxacillin IV/8 weeks of amoxicillin-clavulanate	Recovery/Recovery	None/None	API 20 Strep system, 16 S rDNA gene sequencing
Lemaître et al., 2008	M, 79	Hypertension, intermittent claudication	Plantar phlegmon	Curative debridement and drainage of phlegmon	N.A.	*Klebsiella oxytoca*,* Bacteroides fragilis*	VITEK 2 GP card identification system, 16 S rDNA gene sequencing
McNicholas et al., 2011	M, 75	N.A.	Infection of implantable cardiac device	Flucloxacillin and benzylpenicillin IV (3 days) then vancomycin and clindamycin IV (14days) and oral amoxicillin and rifampicin (4 weeks)	Recovery	None	BBL CrystalTM System, BD Phoenix TM Automated Microbiology System, 16 S rDNA gene sequencing
Pérez-Jorge et al., 2011	M, 39	Osteochondritis	Prosthetic joint chronic infection	Clindamycin and gentamicin initially, then clindamycin and rifampin for 3 months	Recovery	None	API 20 Strep system, 16 S rDNA gene sequencing
Chow et al., 2013	M, 25/M, 68	PTSD, CAD, colonic polyps	Toe abscess/Inner thigh wound from trauma	SXT, cephalexin/Vancomycin and piperacillintazobactam inpatient; cephalexin outpatient	N.A.	*Staphylococcus aureus/Staphylococcus aureus*	API 20 Strep system and VITEK 2, 16 S rDNA gene sequencing
Stanger et al. 2013	M, 86	Malignant melanoma, congenital thrombocytopenia	Osteomyelitis	Cefuroxime and metronidazole, radical debridement, then amoxicillin/clavulanic acid for 6 weeks	Recovery	Normal skin flora, anaerobic bacteria	N.A.
Park et al., 2014	M, 58	Diabetes and end-stage renal disease	Ulcerative lesion of foot	Piperacillin/tazobactam IV for 3 weeks	Recovery	*Proteus mirabilis*	Matrix-assisted laser desorption time-of-flight mass spectrometry, Vitek2 GP system, 16 S rDNA gene sequencing
Sridhar et al., 2014	M, 83	Hypertension, diabetes, prostate cancer	Brain abscess	Ceftriaxone and metronidazole IV (2 weeks), then oral amoxicillin-clavulanate, then ceftriaxone and metronidazole and vancomycin IV (total 12 weeks)	Recovery	None	Vitek2 system, MALDI-TOF MS, 16 S rDNA gene sequencing
Lotte et al., 2015	M, 79	Heavy smoker, alcoholism, dyslipidemia, hypertension and polyvascular disease	Infective endocarditis	Amoxicillin and gentamicin, then Amoxicillin (4 weeks), mitral valve replacement	Recovery	*Klebsiella oxytoca*,* Bacteroides fragilis*	API 20 Strep system, MALDI-TOF MS, 16 S rDNA gene sequencing
Vergne et al., 2015	M(82%), 61(21–91)	Trophic disorders of the lower limbs (77%), cardiovascular pathology (67%), and diabetes mellitus (51%)	Foot ulcers (79%), bacteremia (3%) and other ulcers (18%)	Amoxicillinclavulanate (28%), fluoroquinolones (24%), and third-generation cephalosporins (21%)	NA	*Staphylococcus aureus (62%)*,* Enterobacteria (44%) and Anaerobes (31%)*	MALDI-TOF MS,16 S rDNA gene sequencing
Farid et al., 2017	M, 88	Hypertension, hyperlipidemia, CAD, diastolic HF, paroxysmal AF, COPD, bilateral knee replacements, CKD, and OSA.	Bacteremia, prosthetic valve endocarditis secondary to lower extremity cellulitis	4 weeks of intravenous ceftriaxone. Cardiothoracic surgery and 4-week course of intravenous ceftriaxone at readmission.	Recovery	None	MALDI-TOF MS, leucine amino peptidase and pyrrolidonyl arylamidase test
Li et al., 2021	M, 66	Coronary heart disease, heart failure, hypertension, and type 2 diabetes mellitus	Diabetic foot	Clindamycin and debridement, cefotiam and metronidazole after operation. VSD procedures	Recovery	None	MALDI-TOF-MS, 16 S rRNA gene sequencing
Mouro et al., 2021	M, 17	Cholesteatoma	Mastoiditis complicated by intracranial empyema	Oral cefuroxime and topical ofloxacin. Ceftazidime 2 g tid, metronidazole 1 g bid, and vancomycin 1 g bid (4 weeks). Debridement and IV ciprofloxacin (with metronidazole). Oral ciprofloxacin and metronidazole.	Recovery	*Porphyromonas assacharolitica*,* Bacteroides ovatus*,* B. fragilis*	Mass spectrometry (Vitek MS, bioMérieux, with Vitek MS comprehensive CE- marked database)

Note: N.A. means Not Available, not mentioned in original article. Abbreviation: CAD, coronary artery disease. PTSD, post-traumatic stress disorder. HF, heart failure. AF, atrial fibrillation. COPD, chronic obstructive pulmonary disease. CKD, chronic kidney disease. OSA, obstructive sleep apneoa. VSD, vacuum sealing drainage. Tid, three times per day. Bid, twice per day. IV, intravenous

This report details China’s first documented case of a brain abscess linked to tympanitis caused by *H. kunzii*, marking the fourth known intracranial infection by this bacterium. Previous reports demonstrate variable clinical trajectories. Sridhar et al. (2014) achieved cure through 12-week antibiotic monotherapy for a solitary frontal lobe abscess [18]. A 2020 conference report by Ishak et al. described transient radiological improvement with ceftriaxone followed by fatal deterioration within 4 days [21]. The most recent case (2023) involved mastoiditis-associated polymicrobial abscess requiring combined surgical and antimicrobial intervention [28]. It is noteworthy that the case presented in this study also involved polymicrobial anaerobic co-infection and otogenic origin. However, this case uniquely demonstrates tympanic cavity involvement preceding intracranial spread, expanding the recognized pathophysiology of *H. kunzii* neuro-invasion. The inference of *H. kunzii* as the primary pathogen based on semi-quantitative culture predominance requires cautious interpretation in this polymicrobial context. Potential synergistic interactions between *H. kunzii* and co-infecting anaerobes remain speculative and warrant validation through future metagenomic and co-culture studies.

**Fig. 2:**
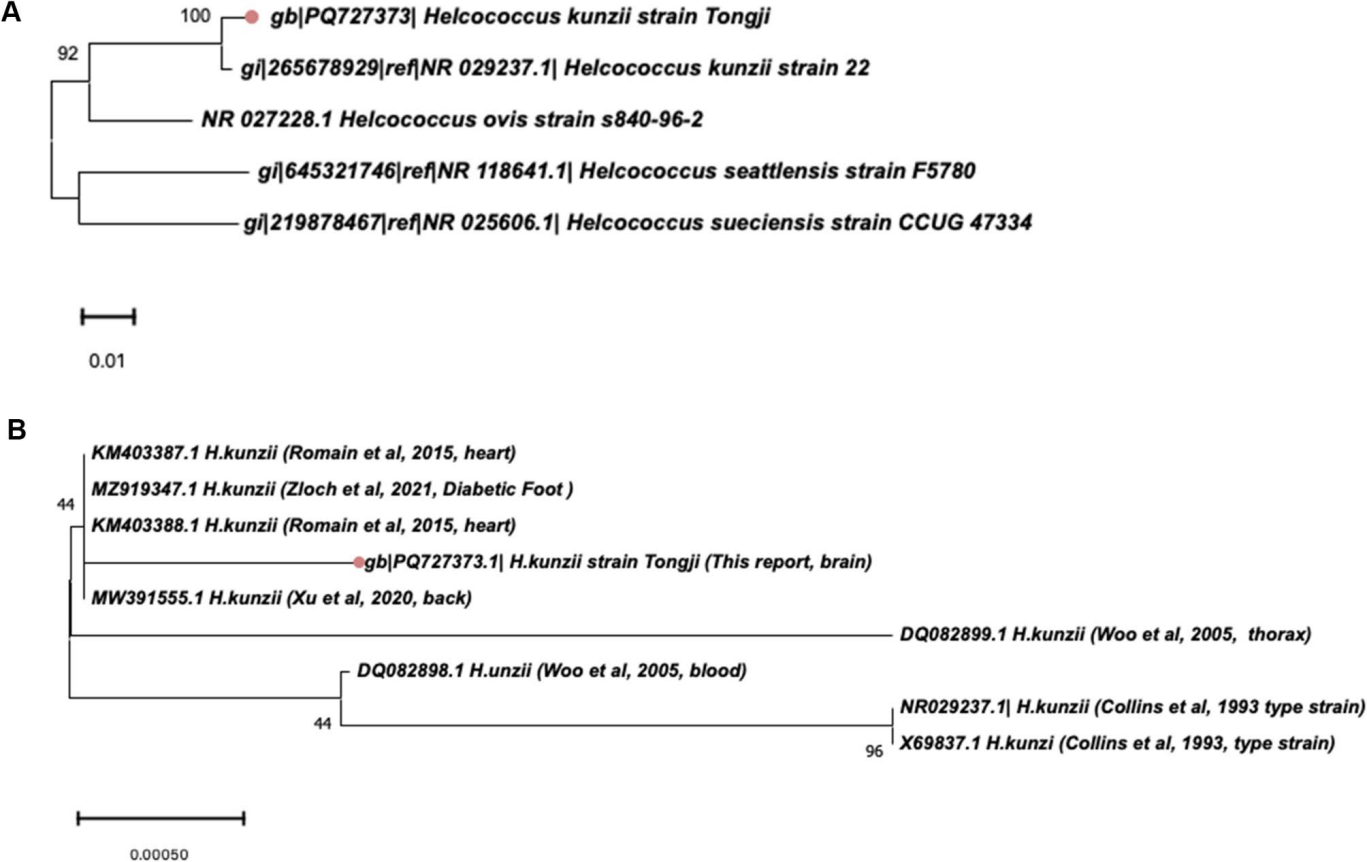
**Phylogenetic tree of** ***Helcococcus kunzi*** (**A**) Phylogenetic tree of *Helcococcus kunzi* and other *Helcococcus* genus. Other compared *Helcococcus* genus included *Helcococcus ovis*, *Helcococcus sueciensis* and *Helcococcus seattlensis*, and only type strains were focused. There was no 16 S rRNA gene sequence of *Helcococcus massiliensis* by Sanger sequencing submitted on GenBank. The length of 16 S rRNA gene sequence of *Helcococcus pyogenica* on the GenBank is only nearly 500 bp, which is not used for comparison. The lengths of all sequences included in the Phylogenetic tree are almost 1400 bp. (**B**) Phylogenetic tree of *H.kunzi* in this report and the *H.kunzi* isolates published by other literature

Accurate identification of *H. kunzii* remains challenging due to biochemical profiling inconsistencies across studies. While conventional API system (API20 STREP, bioMérieux, France) exhibit documented misidentification rates frequently confusing *H. kunzii* with *Aerococcus spp.* [1, 8, 26], our VITEK 2 compact system analysis achieved reliable species-level discrimination (94% confidence). As described in Supplementary Table 1,** H**. *kunzii* consistently tested positive for pyrrolidonyl arylamidase and negative for leucine aminopeptidase. Nevertheless, discrepancies were observed in the biochemical phenotyping, specifically regarding β-galactosidase, maltose, lactose, and ribose outcomes, when comparing our isolate to previously documented strains [5, 9, 15–17, 20, 24, 25, 29]. The MALDI-TOF MS successfully identified our strain at the species level. To corroborate the identification, we performed 16 S rRNA sequencing and submitted the sequence to GenBank under the accession number PQ727373. Our 16 S sequence showed 31-bp divergence from the Collins et al. type strain (NR029237) [1]. Phylogenetic reconstruction using MEGA11 (1400-bp alignment; the original sequence data can be found in Additional File 2) confirmed close clustering with *H. kunzii* (Fig. 2A**)**, while demonstrating distinct differences from other species within the *Helcococcus* genus. Although minor variations are observed at the species level in comparison to other strains in GenBank, the phylogenetic analysis of *H. kunzii* does not indicate significant clustering based on the infection site (refer to Fig. 2B). The present paucity of gene sequences within the database necessitates the continued accumulation of data to draw definitive conclusions.

Supplementary Table 2 summarizes published AST profiles for *H. kunzii*. The pathogen generally demonstrates consistent susceptibility to β-lactams (penicillin, amoxicillin, cephalosporins), quinolones, and glycopeptides [18, 24], supporting Infectious Diseases Society of America's recommendations prioritizing β-lactams as first-line therapy for intracranial infections [30]. This pharmacological profile explains the radiographic resolution observed after ceftriaxone dose escalation in our case, where initial treatment failure likely resulted from suboptimal blood-brain barrier penetration rather than microbial resistance. However, emerging resistance patterns warrant vigilance. Reduced susceptibility to aminoglycosides, sulfonamides, nitroimidazoles, macrolides, and lincosamides has been documented in specific strains [16, 17, 25, 28, 29]. Our isolate’s resistance to erythromycin and clindamycin aligns with reports of *ermA*-mediated macrolide resistance in *Helcococcus* spp [25]., necessitating avoidance of these agents in empirical regimens. Notably, while Mouro et al. [28] observed penicillin resistance in one strain, our findings and most literature confirm preserved β-lactam susceptibility, suggesting resistance mechanisms may be strain-specific or geographically restricted. This geographical variability necessitates region-specific empirical approaches: in settings reporting β-lactam resistance, initial combination therapy with glycopeptides (e.g., norvancomycin) and carbapenems provides dual coverage against both *H. kunzii* and β-lactamase-producing anaerobes, as implemented in our case. Subsequent de-escalation to β-lactam monotherapy should be guided by confirmed susceptibility, particularly in polymicrobial infections where most of cases require concurrent anaerobic coverage. The therapeutic efficacy of norvancomycin in our patient despite in vitro β-lactam susceptibility underscores the complexity of polymicrobial infection management. This approach provided dual coverage against β-lactamase-producing anaerobes while maintaining antimicrobial activity for *H. kunzii*, illustrating the critical role of AST-guided combinatorial therapy in mixed infections. These observations collectively reinforce the imperative for continuous resistance surveillance and pharmacokinetic optimization in treating *H. kunzii* infections.

In conclusion, this study reports the first documented case of a brain abscess secondary to tympanitis caused by *H. kunzii* in China. Together with accumulating evidence of its neuroinvasive potential, these findings position *H. kunzii* as an emerging etiological agent requiring clinical vigilance. For accurate identification amidst its heterogeneous biochemical characteristics, MALDI-TOF MS and 16 S rRNA sequencing should be prioritized in diagnostic workflows.

## Electronic supplementary material

Below is the link to the electronic supplementary material.

Supplementary Material 1

Supplementary Material 2

## Data Availability

All data generated or analysed during this study are included within the article [and its additional files]. Sequence data of this organism that support the findings of this study have also been deposited in GenBank database with the accession number PQ727373.

## Abbreviations

CT: Computed tomography
MRI: Magnetic resonance imaging
MALDI-TOF: Matrix-assisted laser desorption/ionization time of flight
MS: Mass spectrometry
AST: Antimicrobial susceptibility testing
CLSI: Clinical and Laboratory Standards Institute
