# Supplementary material for: A tympanitis-related brain abscess caused by Helcococcus kunzii in China: a case report and literature review
Source: BMC Infect Dis. 2025 Apr 12;25:516. doi: 10.1186/s12879-025-10895-6 (PMC11993954; doi:10.1186/s12879-025-10895-6)
Supplement: Supplementary file 1 — Supplementary Material 1 [file 12879_2025_10895_MOESM1_ESM.doc]

Part One: *H. kunzii* in this article

> gb|PQ727373| Helcococcus kunzii strain Tongji 16S ribosomal RNA gene, partial sequence

TGAAGGGCGCGTCTTACCATGCAGTTGACGAGAATTTTTCAGTTGATTTCTTCGGAATGAAACGGAAAAAGGAAAGTAGCGAACGGGTGAGTAACACGTGAGAAACCTGCCTTTCACAAAGGGATAGCCTCGGGAAACCGGGATTAATACCTTATGATACATTAATATCGCATGATGTAAATGTTAAAGAATTTCGGTGAAAGATGGTCTCGCGTCTGATTAGCTAGTTGGTAAGGTAGTGGCTTACCAAGGCAACGATCAGTAGCCGGATTGAGAGGTTGAACGGCCACACTGGAACTGAGACACGGTCCAGACTCCTACGGGAGGCAGCAGTGGGGAATTTTGCACAATGGGGGAAACCCTGATGCAGCGACGCCGCGTGAACGATGAAGGTCTTCGGATTGTAAAGTTCTGTCCTTAGCGAAGATAATGACAGTAGCTAAGAAGCAAGCCCTGGCTAAATACGTGCCAGCAGCCGCGGTAATACGTATGGGGCAAGCGTTGTCCGGAATTATTGGGCGTAAAGGGTATGTAGGCGGTTAATTAAGTCTGAATTTAAAGGCTGTGGCTCAACCACGGTTCGGTTTAGAAACTGGTTAACTTGAGTAGATGAGGGGGAAGTGGAATTCCATGTGTAGCGGTGAAATGCGTAGATATATGGAGGAACACCAGTGGCGAAGGCGACTTTCTAGAATCTAACTGACGCTGAGATACGAAGGCGTGGGTAGCAAACAGGATTAGATACCCTGGTAGTCCACGCAGTAAACGATGAGTGCTAGTTGTTGGGAGTCAAATCTCAGTGACGCAGCTAACGCATTAAGCACTCCGCCTGGGGAGTACGTACGCAAGTATGAAACTCAAAGGAATTGACGGGGACCCGCACAAGCAGCGGAGCATGTGGTTTAATTCGAAGCAACGCGAAGAACCTTACCAAGGCTTGACATATACAGGAATATCCTAGAGATAGGATAGTCTTTTCGGAGACTTGTATACAGGTGGTGCATGGTTGTCGTCAGCTCGTGTCGTGAGATGTTGGGTTAAGTCCCGTAACGAGCGCAACCCCTATCTTTAGTTACTAGCGAGTAAAGTCGAGGACTCTAGAGAGACTGCCGGTGATAAACCGGAGGAAGGTGGGGATGACGTCAAATCATCATGCCCTATATGTCTTGGGCTACACACGTGCTACAATGGTCTGAACAAAGCGCAGCTACCTCGTGAGAGCAAGCGAATCGCATAAAACAGATCTCAGTTCGGATTGCAGGCTGCAACTCGCCTGCATGAAGTCGGAGTTGCTAGTAATCGTGGATCAGAACGCCACGGTGAATGCGTTCCCGGGTCTTGTACACACCGCCCGTCACACCATGGGAGTTGGCAATACCCGAAGTCGTCGAGCTAACCGTCAAGGAGGCAGACGCCGAAGTAGTCATGCC

Part Two: type strains

>gi|645321746|ref|NR_118641.1| Helcococcus seattlensis strain F5780 16S ribosomal RNA gene, partial sequence

TTTTGGAGAGTTTGATCCTGGCTCAGGACGAACGCTGGCGGCGTGCTTAACACATGCAAGTTGAACGAGA

ATTTTTYTAGAGAWTTCTTCGGAATGATCCATAGAAAAAGGAAAGTAGCGAACGGGTGAGTAACACGTGAGAAACCTGCCTTTCACAAAGGGATAGCTTCGGGAAACCGGAATTAATACCTTATGACACATAARTATCGC

ATGATAAATATGTTAAAGAATTTCGGTGAAAGATGGTCTCGCGTCTGATTAGCTAGATGGTGGGGTAATG

GCCTACCATGGCGATGATCAGTAGCCGGATTGAGAGGTTGAACGGCCACACTGGAACTGAGACACGGTCCAGACTCCTACGGGAGGCAGCAGTGGGGAATTTTGCACAATGGGGGAAACCCTGATGCAGCGACGCCGCGTGAATGATAGAAGGCCTTCGGGTTGTAAAATTCTGTCCTTAGTGAAGATAATGACAGTAACTAAGAAGCAAGCCCTGGCTAAATACGTGCCAGCAGCCGCGGTAATACGTATGGGGCAAGCGTTGTCCGGAATTATTGGGCGTAAAGGGTACGTAGGCGGTTATTTAAGTCCTAATTAAAAGGCAATGGCTCAACCATTGTAAGGTTAGGAAACTAGATAACTTGAGTAGATGAGGGGAAAGTGGAATTCCATGTGTAGCGGTGAAATGCGTAGATATATGGAGGAACACCTGTGGCGAAGGCGACTTTCTGGAATCAAACTGACGCTGAGGTACGAAGGCGTGGGGAGCAAACAGGATTAGATACCCTGGTAGTCCACGCAGTAAACGATGAGTGCTAGTTGTCGGGAGTCAAATCTCGGTGACGCAGCTAACGCATTAAGCACTCCGCCTGGGGAGTACGTGCGCAAGCATGAAACTCAAAGGAATTGACGGGGACCCGCACAAGCAGCGGAGCATGTGGTTTAATTCGAAGCAACGCGAAGAACCTTACCAAGGCTTGACATATACATGGCCGGTGTAGAGATACACCTTTTCTTCGGAACATGTATACAGGTGGTGCATGGTTGTCGTCAGCTCGTGTCGTGAGATGTTGGGTTAAGTCCCGTAACGAGCGCAACCCCTATCTTTAGTTGCTAGCAGGTAAAGCTGAGAACTCTAGAGAGACTGCCGGTGATAAACCGGAGGAAGGTGGGGATGACGTCAAATCATCATGCCCTATATGTCTTGGGCTACACACGTGCTACAATGGTCTGAACAAAGTGCAGCGAACTCGTGAGAGCAAGCGAATCACATAAAACAGATCTCAGTTCGGATTGTAGGCTGCAACTCGCCTACATGAAGTCGGAGTTGCTAGTAATCGTGGATCAGAATGCCACGGTGAATGCGTTCCCGGGTCTTGTACACACCGCCCGTCACACCATGGAAGTTGGCAATACCCGAAGCCGTCGAGCTAACCAATAGGAGGCAGACGTCGAAGGTAGGGTCAATAACTGGGGTGAAGTCGTAACAAGGTAGCCGTATCGGAAGGTGCGGCTGGATCACCTCCTTA

>gi|219878467|ref|NR_025606.1| Helcococcus sueciensis strain CCUG 47334 16S ribosomal RNA gene, partial sequence

TTTGATCCTGGCTCAGGACGAACGCTGGCGGCGTGCTTAACACATGCAAGTTGAACGTGATTTTATAGAC

AGATTTCTTCGGAATGACGATTATAAATGAAAGTAGCGAACGGGTGAGTAACACGTGAGAAACCTGCCTT

TCACAAAGGGATAGCCTCGGGAAACCGGGATTAATACCTTATGATACATAGAAATCGCATGATANCTATG

TTAAAGATTTTATCGGTGAAAGATGGTCTCGCGTCTGATTAGCTAGTTGGTGGGGTAAAGGCCTACCAAG

GCGATGATCAGTAGCCGGATTGAGAGGTTGAACGGCCACACTGGAACTGAGACACGGTCCAGACTCCTACGGGAGGCAGCAGTGGGGAATTTTGCACAATGGGGGAAACCCTGATGCAGCGACGCCGCGTGTACGATGAAGATCTTCGGATTGTAAAGTACTGTCCTTGGTGAAGATAATGACTGTAACCAAGAAGCAAGCCCTGGCTAAATACGTGCCAGCAGCCGCGGTAATACGTATGGGGCAAGCGTTGTCCGGAATTATTGGGCGTAAAGGGTACGTAGGCGGTTATTTAAGTCTAAATTAAAAGGCATTGGCTCAACCAATGTAAGGTTTAGATACTGGATAACTTGAGTAGATGAGGGGAAAGTGGAATTCCATGTGTAGCGGTGAAATGCGTAGATATATGGAGGAACACCAATGGCGAAGGCAACTTTCTGGAATCAAACTGACGCTGAGGTACGAAGGCGTGGGGAGCAAACAGGATTAGATACCCTGGTAGTCCACGCAGTAAACGATGAGTGCTAGATGTTGGGAGTCAAATCTCAGTGTCGCAGCTAACGCAATAAGCACTCCGCCTGGGGAGTACGTACGCAAGTATGAAACTCAAAGGAATTGACGGGGACCCGCACAAGCAGCGGAGCATGTGGTTTAATTCGAAGCAACGCGAAGAACCTTACCAAGGCTTGACATATACATGAAAATCCTAGAGATAGGATCCTCTCTTCGGAGACGTGTATACAGGTGGTGCATGGTTGTCGTCAGCTCGTGTCGTGAGATGTTGGGTTAAGTCCCGTAACGAGCGCAACCCTCGTCTTTAGTTACCAGCAAGTAAAGTTGGGGACTCTAGAGATACTGCCGGTGATAAACCGGAGGAAGGTGGGGATGACGTCAAATCATCATGCCCTATATGTCTTGGGCTACACACGTGCTACAATGGTCTGAACAAAGCGCAGCGAACTCGTGAGGGTAAGCAAATCGCAGAAAACAGATCTCAGTTCGGATTGTAGGCTGCAACTCGCCTACATGAAGTCGGAGTTGCTAGTAATCGTGGATCAGAATGCCACGGTGAATGCGTTCCCGGGTCTTGTACACACCGCCCGTCACACCATGGGAGTTGGCAATACCCGAAGTCGTCGAGCTAACCTTTTAGGAGGCAGACGCCGAAGGTAGGGTCAATAACTGGGGTGAAGTCGTAACAAGGTAGCCGTATCGGAAGGTGCGGCTGGATC

>gi|265678929|ref|NR_029237.1| Helcococcus kunzii strain 22 16S ribosomal RNA gene, partial sequence

GAGAGTTTGATCCTGGCTCAGGACGAACGCTGGCGGCGTGCTTAACACATGCAAGTTGAACGAGAATTTT

TCAGTTGATTTCTTCGGAATGAAACCGAAAANGGAAAGTAGCGAACGGGTGAGTAACACGTGAGAAACCTGCCTTTCACAAAGGGATAGCCTCGGGAAACCGGGATTAATACCTTATGATACATTAATATCGCATGATGTAAATGATGTAAATGTTAAAGAATTTCGGTGAAAGATGGTCTCGCGTCTGATTAGCTAGTTGGTAAGGTACTGGCTTACCAAGGCAACGATCAGTAGCCGGATTGAGAGGTTGAACGGCCACACTGGAACTGAGACACGGTCCAGACTCCTACGGGAGGCAGCAGTGGGGAATTTTGCACAATGGGGGAAACCCTGATGCAGCGACGCCGCGTGAACGATGAAGGTCTTCGGATTGTAAAGTTCTGTCCTTAGCGAAGATAATGACAGTAGCTAAGAAGCAAGCCCTGGCTAAATACGTGCCAGCAGCCGCGGTAATACGTATGGGGCAAGCGTTGTCCGGAATTATTGGGCGTAAAGGGTANGTAGGCGGTTAATTAAGTCTGAATTTAAAGGCTGTGGCTCAACCACGGTTCGGTTTAGAAAACTGGTTAACTTGAGTAGATGAGGGGAAAGTGGAATTCCATGTGTAGCGGTGAAATGCGTAGATATATGGAGGAACACCAGTGGCGAAGGCGACTTTCTAGAATCTAACTGACGCTGAGATACGAAGGCGTGGGTAGCAAACAGGATTAGATACCCTGGTAGTCCACGCAGTAAACGATGAGTGCTAGTTGTTGGGAGTCAAATCTCAGTGACGCAGCTAACGCATTAAGCACTCCGCCTGGGGAGTACGTACGCAAGTATGAAACTCAAAGGAATTGACGGGGACCCGCACAAGCAGCGGAGATGTGGTTTAATTCGAAGCAACGCGAAGAACCTTACCAAGGCTTGAAGGGANATCCTAGAGATAGGATAGTCTTTTCGGAGACTTGTATACAGGTGGTGCATGGTTGTCGTCAGCTCGTGTCGTGAGATGTTGGGTTAAGTCCCGTAACGAGCGCAACCCCTATCTTTAGTTACTAGCGAGTAAAGTCGAGGACTCTAGAGAGACTGCCGGTGATAAACCGGAGGAAGGTGGGGATGACGTCAAATCATCATGCCCTATATGTCTTGGGCTACACACGTGCTACAATGGTCTGAACAAAGCGTAGCTACCTCGTGAGAGCAAGCGAATCGCATAAAACAGATCTCAGTTCGGATTGCAGGCTGCAACTCGCCTGCATGAAGTCGGAGTTGCTAGTAATCGTGGATCAGAACGCCACGGAGAATGCGTTCCCGGGTCTTGTACACACCGCCCGTCACACCATGGGAGTTGGCAATACCCGAAGTCGTCGAGCTAACCGTCAAGGAGGCAGACGCCGAAGGTTGGGTAGAT

>gi|45550016|gb|AY559040.1| Helcococcus pyogenica 16S ribosomal RNA gene, partial sequence

AGAGTTTGATCCTGGCTCAGGACGAACGCTGGCGGCGTGCTTAACACATGCAAGTTGAACGTGATTTTAT

AGACGGATTTCTTCGGAATGACGATTATAAATGAAAGTAGCGAACGGGTGAGTAACACGTGAGAAACCTGCCTTTCACAAAGGGATAGCCTCGGGAAACCGGGATTAATACCTTATGATACATAGAAATCGCATGATAGCTATGTTAAAGATTTTATCGGTGAAAGATGGTCTCGCGTCTGATTAGCTAGTTGGTGGGGTAAAGGCCTACCAAGGCGATGATCAGTAGCCGGATTGAGAGGTTGAACGGCCACACTGGAACTGAGACACGGTCCCAGACTCCTACGGGAGGCAGCAGTGGGGAATTTTGCACAATGGGGGAAACCCTGATGCAGCGACGCCGCGTGTACGATGAAGATCTTCGGATTGTAAAGTACTGTCCTTGGTGAAGATAATGACTGTAACCAAGAAGCAAGCCCTGGCTAAATACGTGCCAGCAGCCGCGGTAA

>NR_027228.1 Helcococcus ovis strain s840-96-2 16S ribosomal RNA, partial sequence

TGGCTCAGGACGAACGCTGGCGGCGTGCTTAACACATGCAAGTTGAACGAGAATTTTTTAATTAATTTCT

TCGGGAAGAGATTAAAGAAGGAAAGTAGCGAACGGGTGAGTAACACGTGAGAAACCTGCCTTTCACAAAG

GGATAGCCTCGGGAAACCGGGATTAATACCTTATGACACTTAGATATCGCATGATAATTAAGTTAAAGAA

TTTCGGTGAAAGATGGTCTCGCGTCTGATTAGCTAGTTGGTAAGGTAACGGCTTACCAAGGCGACGATCA

GTAGCCGGATTGAGAGGTTGAACGGCCACACTGGAACTGAGACACGGTCCAGACTCCTACGGGAGGCAGC

AGTGGGGAATTTTGCACAATGGGGGGAACCCTGATGCAGCGACGCCGCGTGAACGATGAAGGTCTTCGGA

TTGTAAAGTTCTGTCCTTAGTGAAGATAATGACTGTAACTAAGAAGCAAGCCCTGGCTAAATACGTGCCA

GCAGCCGCGGTAATACGTATGGGGCAAGCGTTGTCCGGAATTATTGGGCGTAAAGGGTACGTAGGCGGTA

ATTTAAGTCTGAATTTAAAGGCTGTGGCTCAACCATAGTAAGGTTCAGATACTGGATTACTTGAGTAGAT

GAGGGGAAAGTGGAATTCCATGTGTAGCGGTGAAATGCGTAGATATATGGAGGAACACCTGTGGCGAAGG

CGACTTTCTGGAATCTAACTGACGCTGAGGTACGAAGGCGTGGGGAGCAAACAGGATTAGATACCCTGGT

AGTCCACGCAGTAAACGATGAGTGCTAGTTGTCGGGAGTCAAATCTCGGTGACGCAGCTAACGCATTAAG

CACTCCGCCTGGGGAGTACGTACGCAAGTATGAAACTCAAAGGAATTGACGGGGACCCGCACAAGCAGCG

GAGCATGTGGTTTAATTCGAAGCAACGCGAAGAACCTTACCAAGGCTTGACATATACAGGGATATACTAG

AGATAGTATAGTTTCTTCGGAAACTTGTATACAGGTGGTGCATGGTTGTCGTCAGCTCGTGTCGTGAGAT

GTTGGGTTAAGTCCCGTAACGAGCGCAACCCTTATCTTTAGTTACCAGCATTTCGGATGGGGACTCTAGA

GAGACTGCCGGTGATAAACCGGAGGAAGGTGGGGATGACGTCAAATCATCATGCCCTATATGTCTTGGGC

TACACACGTGCTACAATGGTCTGAACAAAGCGCAGCTACCTCGTGAGAGCAAGCGAATCGCATAAAACAG

ATCTCAGTTCGGATTGTAGGCTGCAACTCGCCTACATGAAGTCGGAGTTGCTAGTAATCGTGGATCAGAA

CGCCACGGTGAATGCGTTCCCGGGTCTTGTACACACCGCCCGTCACACCATGGGAGTTGGCAATACCCGA

AGCCGTCGAGCTAACCGTTAGGA

Part three：other *H. kunzii*

>MW391555.1 Helcococcus kunzii strain ID1 16S ribosomal RNA gene, partial sequence

GCAGTTGACGAGAATTTTTCAGTTGATTTCTTCGGAATGAAACGGAAAAAGGAAAGTAGCGAACGGGTGA

GTAACACGTGAGAAACCTGCCTTTCACAAAGGGATAGCCTCGGGAAACCGGGATTAATACCTTATGATAC

ATTAATATCGCATGATGTAAATGTTAAAGAATTTCGGTGAAAGATGGTCTCGCGTCTGATTAGCTAGTTG

GTAAGGTAGTGGCTTACCAAGGCAACGATCAGTAGCCGGATTGAGAGGTTGAACGGCCACACTGGAACTG

AGACACGGTCCAGACTCCTACGGGAGGCAGCAGTGGGGAATTTTGCACAATGGGGGAAACCCTGATGCAG

CGACGCCGCGTGAACGATGAAGGTCTTCGGATTGTAAAGTTCTGTCCTTAGCGAAGATAATGACAGTAGC

TAAGAAGCAAGCCCTGGCTAAATACGTGCCAGCAGCCGCGGTAATACGTATGGGGCAAGCGTTGTCCGGA

ATTATTGGGCGTAAAGGGTATGTAGGCGGTTAATTAAGTCTGAATTTAAAGGCTGTGGCTCAACCACGGT

TCGGTTTAGAAACTGGTTAACTTGAGTAGATGAGGGGAAAGTGGAATTCCATGTGTAGCGGTGAAATGCG

TAGATATATGGAGGAACACCAGTGGCGAAGGCGACTTTCTAGAATCTAACTGACGCTGAGATACGAAGGC

GTGGGTAGCAAACAGGATTAGATACCCTGGTAGTCCACGCAGTAAACGATGAGTGCTAGTTGTTGGGAGT

CAAATCTCAGTGACGCAGCTAACGCATTAAGCACTCCGCCTGGGGAGTACGTACGCAAGTATGAAACTCA

AAGGAATTGACGGGGACCCGCACAAGCAGCGGAGCATGTGGTTTAATTCGAAGCAACGCGAAGAACCTTA

CCAAGGCTTGACATATACAGGAATATCCTAGAGATAGGATAGTCTTTTCGGAGACTTGTATACAGGTGGT

GCATGGTTGTCGTCAGCTCGTGTCGTGAGATGTTGGGTTAAGTCCCGTAACGAGCGCAACCCCTATCTTT

AGTTACTAGCGAGTAAAGTCGAGGACTCTAGAGAGACTGCCGGTGATAAACCGGAGGAAGGTGGGGATGA

CGTCAAATCATCATGCCCTATATGTCTTGGGCTACACACGTGCTACAATGGTCTGAACAAAGCGCAGCTA

CCTCGTGAGAGCAAGCGAATCGCATAAAACAGATCTCAGTTCGGATTGCAGGCTGCAACTCGCCTGCATG

AAGTCGGAGTTGCTAGTAATCGTGGATCAGAACGCCACGGTGAATGCGTTCCCGGGTCTTGTACACACCG

CCCGTCACACCATGGGAGTTGGCAATACCCGAAGTCGTCGAGCTAACCGTCAAGGAGG

>KM403388.1 Helcococcus kunzii strain 140220302601 16S ribosomal RNA gene, partial sequence

GACGAACGCTGGCGGCGTGCTTAACACATGCAAGTTGAACGAGAATTTTTCAGTTGATTTCTTCGGAATG

AAACCGAAAAAGGAAAGTAGCGAACGGGTGAGTAACACGTGAGAAACCTGCCTTTCACAAAGGGATAGCC

TCGGGAAACCGGGATTAATACCTTATGATACATTAATATCGCATGATGTAAATGTTAAAGAATTTCGGTG

AAAGATGGTCTCGCGTCTGATTAGCTAGTTGGTAAGGTAGTGGCTTACCAAGGCAACGATCAGTAGCCGG

ATTGAGAGGTTGAACGGCCACACTGGAACTGAGACACGGTCCAGACTCCTACGGGAGGCAGCAGTGGGGAATTTTGCACAATGGGGGAAACCCTGATGCAGCGACGCCGCGTGAACGATGAAGGTCTTCGGATTGTAAAG

TTCTGTCCTTAGCGAAGATAATGACAGTAGCTAAGAAGCAAGCCCTGGCTAAATACGTGCCAGCAGCCGC

GGTAATACGTATGGGGCAAGCGTTGTCCGGAATTATTGGGCGTAAAGGGTATGTAGGCGGTTAATTAAGT

CTGAATTTAAAGGCTGTGGCTCAACCACGGTTCGGTTTAGAAACTGGTTAACTTGAGTAGATGAGGGGAA

AGTGGAATTCCATGTGTAGCGGTGAAATGCGTAGATATATGGAGGAACACCAGTGGCGAAGGCGACTTTC

TAGAATCTAACTGACGCTGAGATACGAAGGCGTGGGTAGCAAACAGGATTAGATACCCTGGTAGTCCACG

CAGTAAACGATGAGTGCTAGTTGTTGGGAGTCAAATCTCAGTGACGCAGCTAACGCATTAAGCACTCCGC

CTGGGGAGTACGTACGCAAGTATGAAACTCAAAGGAATTGACGGGGACCCGCACAAGCAGCGGAGCATGT

GGTTTAATTCGAAGCAACGCGAAGAACCTTACCAAGGCTTGACATATACAGGAATATCCTAGAGATAGGA

TAGTCTTTTCGGAGACTTGTATACAGGTGGTGCATGGTTGTCGTCAGCTCGTGTCGTGAGATGTTGGGTT

AAGTCCCGTAACGAGCGCAACCCCTATCTTTAGTTACTAGCGAGTAAAGTCGAGGACTCTAGAGAGACTG

CCGGTGATAAACCGGAGGAAGGTGGGGATGACGTCAAATCATCATGCCCTATATGTCTTGGGCTACACAC

GTGCTACAATGGTCTGAACAAAGCGCAGCTACCTCGTGAGAGCAAGCGAATCGCATAAAACAGATCTCAG

TTCGGATTGCAGGCTGCAACTCGCCTGCATGAAGTCGGAGTTGCTAGTAATCGTGGATCAGAACGCCACG

GTGAATGCGTTCCCG

>KM403387.1 Helcococcus kunzii strain CIP 103932 16S ribosomal RNA gene, partial sequence

GACGAACGCTGGCGGCGTGCTTAACACATGCAAGTTGAACGAGAATTTTTCAGTTGATTTCTTCGGAATG

AAACCGAAAAAGGAAAGTAGCGAACGGGTGAGTAACACGTGAGAAACCTGCCTTTCACAAAGGGATAGCC

TCGGGAAACCGGGATTAATACCTTATGATACATTAATATCGCATGATGTAAATGTTAAAGAATTTCGGTG

AAAGATGGTCTCGCGTCTGATTAGCTAGTTGGTAAGGTAGTGGCTTACCAAGGCAACGATCAGTAGCCGG

ATTGAGAGGTTGAACGGCCACACTGGAACTGAGACACGGTCCAGACTCCTACGGGAGGCAGCAGTGGGGAATTTTGCACAATGGGGGAAACCCTGATGCAGCGACGCCGCGTGAACGATGAAGGTCTTCGGATTGTAAAG

TTCTGTCCTTAGCGAAGATAATGACAGTAGCTAAGAAGCAAGCCCTGGCTAAATACGTGCCAGCAGCCGC

GGTAATACGTATGGGGCAAGCGTTGTCCGGAATTATTGGGCGTAAAGGGTATGTAGGCGGTTAATTAAGT

CTGAATTTAAAGGCTGTGGCTCAACCACGGTTCGGTTTAGAAACTGGTTAACTTGAGTAGATGAGGGGAA

AGTGGAATTCCATGTGTAGCGGTGAAATGCGTAGATATATGGAGGAACACCAGTGGCGAAGGCGACTTTC

TAGAATCTAACTGACGCTGAGATACGAAGGCGTGGGTAGCAAACAGGATTAGATACCCTGGTAGTCCACG

CAGTAAACGATGAGTGCTAGTTGTTGGGAGTCAAATCTCAGTGACGCAGCTAACGCATTAAGCACTCCGC

CTGGGGAGTACGTACGCAAGTATGAAACTCAAAGGAATTGACGGGGACCCGCACAAGCAGCGGAGCATGT

GGTTTAATTCGAAGCAACGCGAAGAACCTTACCAAGGCTTGACATATACAGGAATATCCTAGAGATAGGA

TAGTCTTTTCGGAGACTTGTATACAGGTGGTGCATGGTTGTCGTCAGCTCGTGTCGTGAGATGTTGGGTT

AAGTCCCGTAACGAGCGCAACCCCTATCTTTAGTTACTAGCGAGTAAAGTCGAGGACTCTAGAGAGACTG

CCGGTGATAAACCGGAGGAAGGTGGGGATGACGTCAAATCATCATGCCCTATATGTCTTGGGCTACACAC

GTGCTACAATGGTCTGAACAAAGCGCAGCTACCTCGTGAGAGCAAGCGAATCGCATAAAACAGATCTCAG

TTCGGATTGCAGGCTGCAACTCGCCTGCATGAAGTCGGAGTTGCTAGTAATCGTGGATCAGAACGCCACG

GTGAATGCGTTCCCG

>DQ082899.1 Helcococcus kunzii clone 2 16S ribosomal RNA gene, partial sequence

CGGCGTGCTTAACACATGCAAGTTGAACGAGAATTTTTCAGTCGATTTCTTCGGAATGAAACCGAAAAAG

GAAAGTAGCGAACGGGTGAGTAACACGTGAGAAACCTGCCTTTCACAAAGGGATAGCCTCGGGAAACCGG

GATTAATACCTTATGATACATTAATATCGCATGATGTAAATGTTAAAGAATTTCGGTGAAAGATGGTCTC

GCGTCTGATTAGCTAGTTGGTAAGGTAGTGGCTTACCAAGGCAACGATCAGTAGCCGGATTGAGAGGTTG

AACGGCCACACTGGAACTGAGACACGGTCCAGACTCCTACGGGAGGCAGCAGTGGGGAATTTTGCACAAT

GGGGGAAACCCTGATGCAGCGACGCCGCGTGAACGATGAAGGTCTTCGGATTGTAAAGTTCTGTCCTTAG

CGAAGATAATGACAGTAGCTAAGAAGCAAGCCCTGGCTAAATACGTGCCAGCAGCCGCGGTAATACGTAT

GGGGCAAGCGTTGTCCGGAATTATTGGGCGTAAAGGGTATGTAGGCGGTTAATTAAGTCTGAATTTAAAG

GCTGTGGCTCAACCACGGTTCGGTTTAGAAACTGGTTAACTTGAGTAGATGAGGGGAAAGTGGAATTCCA

TGTGTAGCGGTGAAATGCGTAGATATATGGAGGAACACCAGTGGCGAAGGCGACTTTCTAGAATCTAACT

GACGCTGAGATACGAAGGCGTGGGTAGCAAACAGGATTAGATACCCTGGTAGTCCACGCAGTAACGATGA

GTGCTAGTTGTTGGGAGTCAAATCTCAGTGACGCAGCTAACGCATTGAGCACTCCGCCTGGGGAGTACGT

ACGCAAGTATGAAACTCAAAGGAATTGACGGGGACCCGCACAAGCACGGAGCATGTGGTTTAATTCGAAG

CAACGCGAAGAACCTTACCAAGGCTTGACATATACAGGAATATCCTAGAGATAGGATAGTCTTTTCGGAG

ACTTGTATACAGGTGGTGCATGGTTGTCGTCAGCTCGTGTCGTGAGATGTTGGGTTAAGTCCCGTAACGA

GCGCAACCCCTATCTTTAGTTACTAGCGAGTAAAGTCGAGGACTCTAGAGAGACTGCCGGTGATAAACCG

GAGGAAGGTGGGGATGACGTCAAATCATCATGCCCTATATGTCTTGGGCTACACACGTGCTACAATGGTC

TGAACAAAGCGTAGCTACCTCGTGAGAGCAAGCGAATCGCATAAAACAGATCTCAGTTCGGATTGCAGGC

TGCAACTCGCCTGCATGAAGTCGGAATTGCTAGTAATCGTGGATCAGAAC

>DQ082898.1 Helcococcus kunzii clone 1 16S ribosomal RNA gene, partial sequence

CACGTGAGAAACCTGCCTTTCACAAAGGGATAGCCTCGGGAAACCGGGATTAATACCTTATGATACATTA

ATATCGCATGATGTAAATGTTAAAGAATTTCGGTGAAAGATGGTCTCGCGTCTGATTAGCTAGTTGGTAA

GGTAGTGGCTTACCAAGGCAACGATCAGTAGCCGGATTGAGAGGTTGAACGGCCACACTGGAACTGAGAC

ACGGTCCAGACTCCTACGGGAGGCAGCAGTGGGGAATTTTGCACAATGGGGGAAACCCTGATGCAGCGAC

GCCGCGTGAACGATGAAGGTCTTCGGATTGTAAAGTTCTGTCCTTAGCGAAGATAATGACAGTAGCTAAG

AAGCAAGCCCTGGCTAAATACGTGCCAGCAGCCGCGGTAATACGTATGGGGCAAGCGTTGTCCGGAATTA

TTGGGCGTAAAGGGTATGTAGGCGGTTAATTAAGTCTGAATTTAAAGGCTGTGGCTCAACCACGGTTCGG

TTTAGAAACTGGTTAACTTGAGTAGATGAGGGGAAAGTGGAATTCCATGTGTAGCGGTGAAATGCGTAGA

TATATGGAGGAACACCAGTGGCGAAGGCGACTTTCTAGAATCTAACTGACGCTGAGATACGAAGGCGTGG

GTAGCAAACAGGATTAGATACCCTGGTAGTCCACGCAGTAAACGATGAGTGCTAGTTGTTGGGAGTCAAA

TCTCAGTGACGCAGCTAACGCATTAAGCACTCCGCCTGGGGAGTACGTACGCAAGTATGAAACTCAAAGG

AATTGACGGGGACCCGCACAAGCAGCGGAGCATGTGGTTTAATTCGAAGCAACGCGAAGAACCTTACCAA

GGCTTGACATATACAGGGATATCCTAGAGATAGGATAGTCTTTTCGGAGACTTGTATACAGGTGGTGCAT

GGTTGTCGTCAGCTCGTGTCGTGAGATGTTGGGTTAAGTCCCGTAACGAGCGCAACCCCTATCTTTAGTT

ACTAGCGAGTAAAGTCGAGGACTCTAGAGAGACTGCCGGTGATAAACCGGAGGAAGGTGGGGATGACGTC

AAATCATCATGCCCTATATGTCTTGGGCTACACACGTGCTACAATGGTCTGAACAAAGCGCAGCTACCTC

GTGAGAGCAAGCGAATCGCATAAAACAGATCTCAGTTCGGATTGCAGGCTGCAACTCGCCTGCATGAAGT

CGGAGTTGCTAGTAATCGTGGATCAGAACGCCACGGTGAATGCGTTCCCGGGTCTTGTACACACCGCCCG

TCACACCATGGGAGTTGGCAATACCCGAAGTCGTCGAGCTATCCGTCAAGCACGC

>MZ919347.1 Helcococcus kunzii strain DFI-7 16S ribosomal RNA gene, partial sequence

CGAGAATTTTTCAGTTGATTTCTTCGGAATGAAACGGAAAAAGGAAAGTAGCGAACGGGTGAGTAACACG

TGAGAAACCTGCCTTTCACAAAGGGATAGCCTCGGGAAACCGGGATTAATACCTTATGATACATTAATAT

CGCATGATGTAAATGTTAAAGAATTTCGGTGAAAGATGGTCTCGCGTCTGATTAGCTAGTTGGTAAGGTA

GTGGCTTACCAAGGCAACGATCAGTAGCCGGATTGAGAGGTTGAACGGCCACACTGGAACTGAGACACGG

TCCAGACTCCTACGGGAGGCAGCAGTGGGGNATTTTGCACAATGGGGGAAACCCTGATGCAGCGACGCCG

CGTGAACGATGAAGGTCTTCGGATTGTAAAGTTCTGTCCTTAGCGAAGATAATGACAGTAGCTAAGAAGC

AAGCCCTGGCTAAATACGTGCCAGCAGCCGCGGTAATACGTATGGGGCAAGCGTTGTCCGGAATTATTGG

GCGTAAAGGGTATGTAGGCGGTTAATTAAGTCTGAATTTAAAGGCTGTGGCTCAACCACGGTTCGGTTTA

GAAACTGGTTAACTTGAGTAGATGAGGGGAAAGTGGAATTCCATGTGTAGCGGTGAAATGCGTAGATATA

TGGAGGAACACCAGTGGCGAAGGCGACTTTCTAGAATCTAACTGACGCTGAGATACGAAGGCGTGGGTAG

CAAACAGGATTAGATACCCTGGTAGTCCACGCAGTAAACGATGAGTGCTAGTTGTTGGGAGTCAAATCTC

AGTGACGCAGCTAACGCATTAAGCACTCCGCCTGGGGAGTACGTACGCAAGTATGAAACTCAAAGGAATT

GACGGGGACCCGCACAAGCAGCGGAGATGTGGTTTAATTCGAAGCAACGCGAAGAACCTTACCAAGGCTT

GAAGGAATATCCTAGAGATAGGATAGTCTTTTCGGAGACTTGTATACAGGTGGTGCATGGTTGTCGTCAG

CTCGTGTCGTGAGATGTTGGGTTAAGTCCCGTAACGAGCGCAACCCCTATCTTTAGTTACTAGCGAGTAA

AGTCGAGGACTCTAGAGAGACTGCCGGTGATAAACCGGAGGAAGGTGGGGATGACGTCAAATCATCATGC

CCTATATGTCTTGGGCTACACACGTGCTACAATGGTCTGAACAAAGCGCAGCTACCTCGTGAGAGCAAGC

GAATCGCATAAAACAGATCTCAGTTCGGATTGCAGGCTGCAACTCGCCTGCATGAAGTCGGAGTTGCTAG

TAATCGTGGATCAGAACGCCACGGTGAATGCGTTCCCGGGTCTTGTACACACCGCCCGTCACACCATGGG

AGTTGGCAATACCCGAAGTCGTCGAGCTAACCGTCAAGGAGGCAGACGCCGAAG

>X69837.1 H.kunzii gene for 16S rRNA, partial

GAGAGTTTGATCCTGGCTCAGGACGAACGCTGGCGGCGTGCTTAACACATGCAAGTTGAACGAGAATTTT

TCAGTTGATTTCTTCGGAATGAAACCGAAAANGGAAAGTAGCGAACGGGTGAGTAACACGTGAGAAACCT

GCCTTTCACAAAGGGATAGCCTCGGGAAACCGGGATTAATACCTTATGATACATTAATATCGCATGATGT

AAATGATGTAAATGTTAAAGAATTTCGGTGAAAGATGGTCTCGCGTCTGATTAGCTAGTTGGTAAGGTAC

TGGCTTACCAAGGCAACGATCAGTAGCCGGATTGAGAGGTTGAACGGCCACACTGGAACTGAGACACGGT

CCAGACTCCTACGGGAGGCAGCAGTGGGGAATTTTGCACAATGGGGGAAACCCTGATGCAGCGACGCCGC

GTGAACGATGAAGGTCTTCGGATTGTAAAGTTCTGTCCTTAGCGAAGATAATGACAGTAGCTAAGAAGCA

AGCCCTGGCTAAATACGTGCCAGCAGCCGCGGTAATACGTATGGGGCAAGCGTTGTCCGGAATTATTGGG

CGTAAAGGGTANGTAGGCGGTTAATTAAGTCTGAATTTAAAGGCTGTGGCTCAACCACGGTTCGGTTTAG

AAAACTGGTTAACTTGAGTAGATGAGGGGAAAGTGGAATTCCATGTGTAGCGGTGAAATGCGTAGATATA

TGGAGGAACACCAGTGGCGAAGGCGACTTTCTAGAATCTAACTGACGCTGAGATACGAAGGCGTGGGTAG

CAAACAGGATTAGATACCCTGGTAGTCCACGCAGTAAACGATGAGTGCTAGTTGTTGGGAGTCAAATCTC

AGTGACGCAGCTAACGCATTAAGCACTCCGCCTGGGGAGTACGTACGCAAGTATGAAACTCAAAGGAATT

GACGGGGACCCGCACAAGCAGCGGAGATGTGGTTTAATTCGAAGCAACGCGAAGAACCTTACCAAGGCTT

GAAGGGANATCCTAGAGATAGGATAGTCTTTTCGGAGACTTGTATACAGGTGGTGCATGGTTGTCGTCAG

CTCGTGTCGTGAGATGTTGGGTTAAGTCCCGTAACGAGCGCAACCCCTATCTTTAGTTACTAGCGAGTAA

AGTCGAGGACTCTAGAGAGACTGCCGGTGATAAACCGGAGGAAGGTGGGGATGACGTCAAATCATCATGC

CCTATATGTCTTGGGCTACACACGTGCTACAATGGTCTGAACAAAGCGTAGCTACCTCGTGAGAGCAAGC

GAATCGCATAAAACAGATCTCAGTTCGGATTGCAGGCTGCAACTCGCCTGCATGAAGTCGGAGTTGCTAG

TAATCGTGGATCAGAACGCCACGGAGAATGCGTTCCCGGGTCTTGTACACACCGCCCGTCACACCATGGG

AGTTGGCAATACCCGAAGTCGTCGAGCTAACCGTCAAGGAGGCAGACGCCGAAGGTTGGGTAGAT
