# Supplementary material for: A tympanitis-related brain abscess caused by Helcococcus kunzii in China: a case report and literature review
Source: BMC Infect Dis. 2025 Apr 12;25:516. doi: 10.1186/s12879-025-10895-6 (PMC11993954; doi:10.1186/s12879-025-10895-6)
Supplement: Supplementary file 2 — Supplementary Material 2 [file 12879_2025_10895_MOESM2_ESM.docx]

**On admission:** a ten-day history of dizziness, headache, and vomiting

**Physical，laboratory，**

**radiological Examinations**

**Surgery: Occipital Lobectomy**

**Antibiotics: intravenous meropenem, 1g, q8h**

**Diagnostic Evaluation:** a suspected tympanitis-related brain abscess

**Nutritional Support and Symptomatic Therapy**

**Diagnose:** abscess infection caused by *Helcococcus kunzii,* *Peptoniphilus harei，Peptostreptococcus lactolyticus*

**Discharge from hospital**

**Return outpatient visit**

**Antibiotics: intravenous ceftriaxone, 3g, qd**

**Antibiotics：intravenous** **norvancomycin 0.8g, bid**

**Supplemental Figure 1. Timeline of diagnostic and therapeutic events after admission.**

**2023-11-26**

**2024-01-09**

**2023-12-05**

**2023-11-23**

**2023-11-20**

**
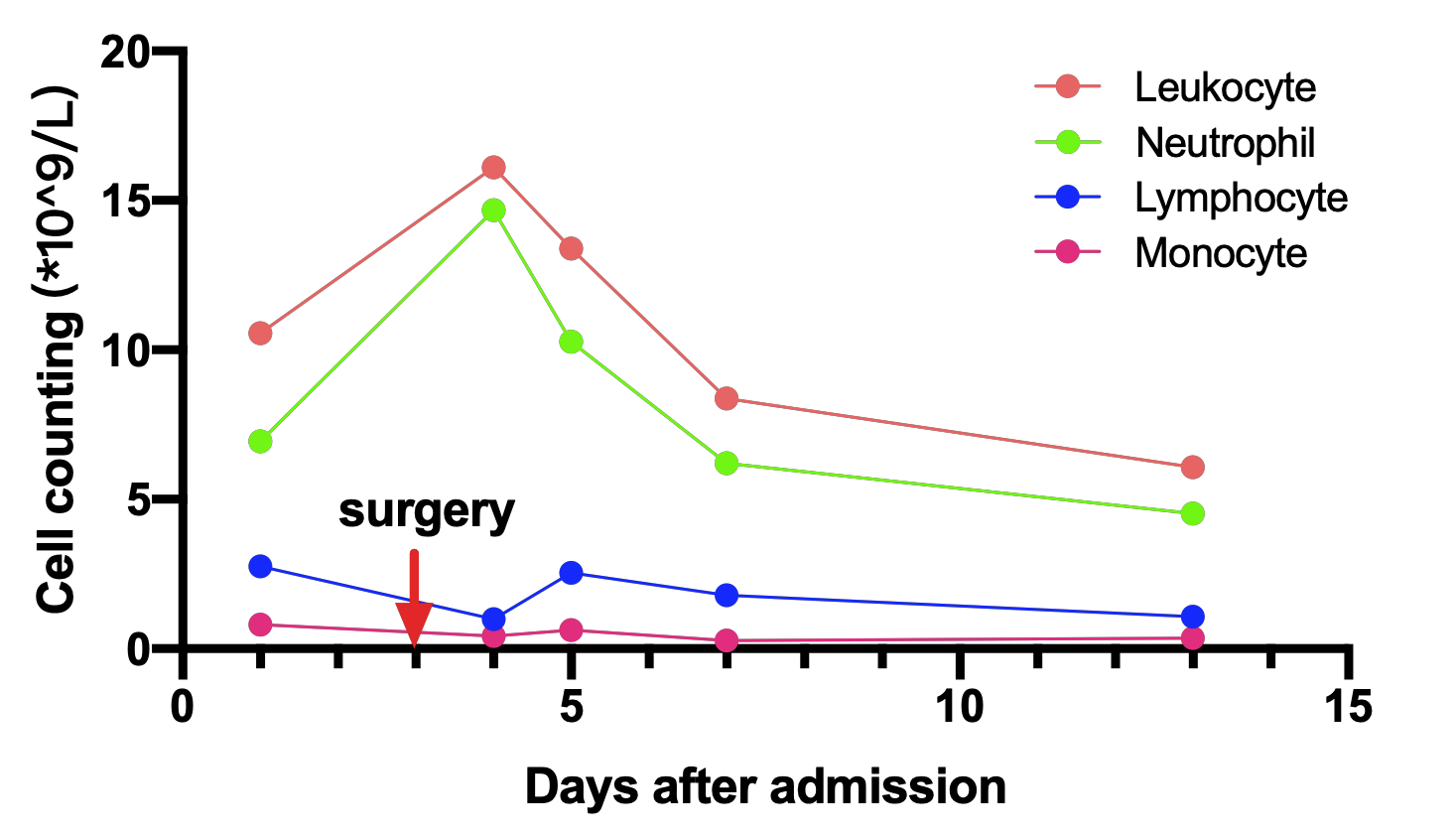
**

**Supplemental Figure 2. Dynamic changes of inflammatory indicators during hospitalization.** Red arrow indicates the time point at which the patient underwent surgery.

**Supplementary Table 1. Biochemical results for** ***H. kunzii* isolate in different studies.**

| Phenotypic characteristic | Peel et al., 1997 | Chagla et al., 1998 | Riegel et al, 2003 | Woo et al., 2005 | Lemaître et al., 2008 | Pérez-Jorge et al., 2011 | Chow et al, 2013 | Park et al., 2014 | Farid et al., 2017 | This report |
| --- | --- | --- | --- | --- | --- | --- | --- | --- | --- | --- |
| β-Galactosidase | - |  |  |  | + |  | + |  |  | + |
| α-Glucosidase | - |  |  |  |  |  |  |  |  | - |
| Pyrrolidonyl arylamidase | + | + | + | + | + | + | + | + | + | + |
| Esculin hydrolysis | + | + |  |  |  | + |  |  |  |  |
| Pyrazinamidase | + |  |  |  |  |  |  |  |  |  |
| Cellobiose | + |  |  |  |  |  |  |  |  |  |
| Glucose | + | + |  |  |  |  |  |  |  |  |
| Maltose | + | + |  |  |  |  |  |  |  | - |
| Lactose | + | + |  |  |  | + | + |  |  | - |
| Trehalose | + | - |  |  |  | + | + |  |  |  |
| Starch | + | + |  |  |  | - | + |  |  |  |
| Glycogen | + | - |  |  |  |  | + |  |  |  |
| Sucrose | - | - |  |  |  |  |  |  |  | - |
| Ribose | - |  |  |  |  |  | + |  |  | - |
| Arabinose | - | - |  |  |  |  |  |  |  |  |
| Mannitol | - | - |  |  |  |  |  |  |  | - |
| Sorbitol | - | - |  |  |  |  |  |  |  | - |
| Inulin | - | + |  |  |  |  | - |  |  |  |
| Raffinose | - | - |  |  |  |  | - |  |  | - |
| Hippurate hydrolysis | - | - | - |  |  |  |  |  |  |  |
| Alkaline phosphatase | - |  |  |  |  | - |  |  |  |  |
| Leucine aminopeptidase | - | - | - | - |  |  | - | - | - |  |
| Arginine dihydrolase | - | - | - |  |  |  |  |  |  |  |
| Gelatin liquefaction | - |  |  |  |  |  |  |  |  |  |
| Nitrate reduction | - |  |  |  |  |  |  |  |  |  |
| Urease | - | - |  |  |  |  |  |  |  |  |
| Voges-Proskauer |  | - |  |  |  |  |  |  |  |  |
| 6.5% NaCl | + | + | + |  |  | + |  |  |  |  |
| Melibiose |  | - |  |  |  |  |  |  |  |  |
| Salicin |  | + |  |  |  |  |  |  |  | + |
| Sorbose |  | - |  |  |  |  |  |  |  |  |
| Acetoin |  |  | - |  |  |  |  |  |  |  |
| D-mannose |  |  |  |  | + |  |  |  |  |  |
| β-galactopyranosidase |  |  |  |  | + | + |  |  |  | + |
| alanine arylamidase |  |  |  |  | + |  |  |  |  | + |
| leucine arylamidase |  |  |  |  | + | - |  |  |  | - |
| β-Glucosidase |  |  |  |  |  | + |  |  |  |  |
| β-Glucuronidase |  |  |  |  |  | - | - |  |  |  |
| amygdalin |  |  |  |  |  |  |  |  |  | + |
| Methyl-β-  D-glucopyranoside |  |  |  |  |  |  |  |  |  | + |

Symbols: +, positive; -, negative.

**Supplementary Table 2.** **Antimicrobial susceptibilities of *Helcococcus kunzii* in different studies.**

| Author | method | Penicillin G | Clindamycin | Vancomycin | Erythromycin | cefotaxime | linezolid | Ceftriaxone | Levofloxacin | Chloramphenicol |
| --- | --- | --- | --- | --- | --- | --- | --- | --- | --- | --- |
| Peel et al., 1997 | E-test | S(0.094) | / | S(0.75) | / | / | / | / | / | / |
| Chagla et al., 1998 | E-test | S (0.25) | S(0.25) | / | R(1.0) | / | / | / | / | / |
| Riegel et al, 2003 | Disk diffusion, E-test | S(0.064) | / | S | R | S(0.5) | / | / | / | / |
| Woo et al., 2005 | Broth macrodilution | S(0.064)/S(0.032) | R(0.5)/S(0.25) | S(0.5)/S(0.5) | R(256)/S(0.25) | S(0.25)/S(0.032) | / | / | / | / |
| Lemaître et al., 2008 | Disk diffusion | S | / | S | / | / | / | / | / | / |
| McNicholas et al., 2011 | N.A. | S(0.008) | / | / | / | / | / | / | / | / |
| Pérez-Jorge et al., 2011 | Disk diffusion | / | S | S | R | / | S | / | / | / |
| Chow et al., 2013 | Sensititre system, E-test, MicroScan system with a MICroSTREP plus panel | 0.25/0.03 | 0.5/0.5 | 0.5/0.5 | 1/256 | 0.12/0.12 | 4/2 | 0.25/0.12 | 1/1 | 16/8 |
| Park et al., 2014 | E-test | S | R | / | R | / | / | / | / | / |
| Sridhar et al., 2014 | Disk diffusion, E-test | S (0.003) | / | S(0.25) | / | / | / | S(0.016) | / | / |
| Lotte et al., 2015 | E-test | S (0.016) | S (0.032) | S (0,38) | / | S (0.016) | / | / | / | / |
| Farid et al., 2017 | E-test | S | / | S | / | / | / | S(0.5) | / | / |
| Li et al., 2021 | Disk diffusion | 36 | 6 | 36 | 6 | / | / | / | / | / |
| Mouro et al., 2021 | Disk diffusion | R | R | S | R | / | S | / | / | / |
| This report | Disk diffusion, E-test | S(0.016) | R(6) | S(28) | R(6) | S(28) | S(28) | S(0.094) | S(26) | S(22) |

Note: N.A means Not Available, the method of AST was not mentioned. / denotes this drug was not tested.
